# Polystyrene Microplastics Induced Ovarian Toxicity in Juvenile Rats Associated with Oxidative Stress and Activation of the PERK-eIF2α-ATF4-CHOP Signaling Pathway

**DOI:** 10.3390/toxics11030225

**Published:** 2023-02-27

**Authors:** Wanzhen Wang, Jiafu Guan, Yueying Feng, Shanji Liu, Yu Zhao, Yuanyuan Xu, Hengyi Xu, Fen Fu

**Affiliations:** 1 The Second Affiliated Hospital of Nanchang University, 235 Nanjing East Road, Nanchang 330047, China; 2 State Key Laboratory of Food Science and Technology, Nanchang University, 235 Nanjing East Road, Nanchang 330047, China

**Keywords:** polystyrene microplastics, juvenile, ovarian toxicity, oxidative stress, endoplasmic reticulum stress

## Abstract

Numerous reports confirm that microplastics exposure could induce reproductive toxicity in mammals. However, the effects of microplastics exposure during juveniles on ovarian apoptosis through oxidative and endoplasmic reticulum (ER) stresses remains unclear, which is the focus of our study. In the present study, female rats (4 weeks old) were exposed to polystyrene microplastics (PS-MPs, 1 μm) at different dosages (0, 0.5, and 2.0 mg/kg) for 28 days. Findings revealed that 2.0 mg/kg of PS-MPs distinctly increased the atretic follicle ratio in the ovary and dramatically reduced the serum levels of estrogen and progesterone. Additionally, the oxidative stress indicators declined, including the activity of superoxide dismutase and catalase, whereas the malondialdehyde content in the ovary was considerably enhanced in the 2.0 mg/kg PS-MPs group. Furthermore, the expressions of genes related to ER stress (PERK, eIF2α, ATF4, and CHOP) and apoptosis were remarkably elevated in the 2.0 mg/kg PS-MPs group compared with those in the control group. We found that PS-MPs induced oxidative stress and activated the PERK-eIF2α-ATF4-CHOP signaling pathway in juvenile rats. Moreover, with the oxidative stress inhibitor N-acetyl-cysteine and eIF2α dephosphorylation blocker Salubrinal treatment, ovarian damage induced by PS-MPs was repaired and associated enzyme activities were improved. Overall, our results indicated that PS-MPs exposure induced ovarian injury associated with oxidative stress and activation of the PERK-eIF2α-ATF4-CHOP signaling pathway in juvenile rats, providing new prospects for assessing the health risks of children exposed to microplastics.

## 1. Introduction

Microplastics (MPs) are smaller than 5 mm in size and are developed from plastic debris through the long-term effects of water, ultraviolet radiation, and biological degradation [1]. According to estimates, the accumulation of MPs in the soil is 4 to 23 times greater than in the ocean, posing a major threat to terrestrial ecosystems [2]. MPs have become typical environmental contaminants and can be found in a wide variety of ecological settings, biological species, human living habitats, and even ordinary necessities, such as shampoos, toothpaste, and cosmetics [3,4]. Moreover, emerging evidence indicates that humans are ubiquitously exposed to MPs [5] mainly through oral ingestion, inhalation, and skin contact. Previous research has established that MPs may build in the digestive organs [6], the blood [7], and the placenta [8], posing a health concern owing to their complicated chemical characteristics. MPs have been linked to a high risk of metabolic disruption, neurotoxicity, and cancer in humans [9]. Current research suggests that MPs could cause dose-dependent microstructural and functional ovarian damage, which is mediated by pro-oxidant and pro-inflammatory pathways. These pathways affect genes, molecular effectors, and hormones that regulate folliculogenesis [10]. Thus, MPs may affect human reproduction, requiring a full assessment of the harm caused by MPs to the human reproductive system. Pregnancy, infancy, and puberty are highly vulnerable periods in which the sensitivity to environmental pollution is high [11,12]. Nevertheless, knowledge about the reproductive toxicity induced by MPs exposure at different stages of human growth, especially during childhood, is limited.

Children’s special behaviors, such as crawling, hand–mouth activity, playing with toys, and touching personal products, increase their exposure to MPs [13]. Researchers recently found that the exposure of infants and children to MPs is indeed greater than that of adults [14]. The effects on reproduction are one of the main concerns when considering the effects of MPs on children’s health [15]. Children’s reproductive systems are immature; thus, juvenile reproductive toxicity can eventually lead to infertility [16]. A previous study demonstrated that MPs exposure (five weeks) in a hypothetical scenario may pose an irreversible endocrine disturbance with particular indicators of reproductive toxicity in mammals [17]. The ovaries’ hormone secretion contributes to maintaining pubertal development and growth. Mice exposed to MPs developed inflammation of the ovaries and experienced reduced-quality oocytes [18]. MPs trigger reproductive damage in mice by the peroxidation and amplification of the P38 MAPK signal transduction [19]. These investigations prove that MPs can cause oxidative stress, which ultimately leads to reproductive damage [20]. It is postulated that oxidative stress is one of the key processes causing female infertility [21]. Of note, Haddadi et al. recently published that 5 μm MPs exposure by oral gavage was detected in the duodenum and ovary in rats, leading to defective ovarian function via oxidative stress [22]. The abnormal production of free radicals (ROS) might contribute to the buildup of unfolded or misfolded peptides in the endoplasmic reticulum lumen [23]. However, if the system is overwhelmed with the accumulation of misfolded proteins, the unfolded protein response (UPR) is provoked to trigger ER stress. Chronic ER stress mediates apoptotic signaling. Under pathological conditions, the protein kinase R-like ER kinase (PERK) signaling pathway was activated and dissociated from the ER chaperone immunoglobulin protein (BIP) which is the initiator of ER stress, exacerbating cellular apoptosis [24]. PERK is activated and directly phosphorylates the eukaryotic initiation factor (eIF2α), which in turn activates the transcription factor the C/EBP homologous protein (CHOP) mainly through the activated transcription factor 4 (ATF4) [25]. Extensive studies have demonstrated that ER stress exerts a pivotal role in the development of the ovary and involves multiple ovarian diseases [26]. Nevertheless, the mechanism related to ER stress-activated by MPs is rarely articulated. The Female Pubertal Assay serves as an in vivo screening assay to help identify chemicals or mixtures that have the potential to interact with the endocrine system, by identifying effects on pubertal development and thyroid function in the intact juvenile/peripubertal female rat. This assay is capable of detecting anti-thyroid, estrogenic, or anti-estrogenic chemicals, or agents which alter pubertal development [27]. Consequently, the following contents will elucidate the ovarian apoptosis in detail induced by MPs exposure through the oxidative and ER stresses in juvenile rats.

Polystyrene MPs (PS-MPs) were widely used to study toxic effects in mammals. Therefore, our experiment aimed to assess the ovarian poisoning from PS-MPs in juvenile female rats and identify the inherent risks of a 28-day exposure to PS-MPs. We assumed that MPs could induce ovary injury in juvenile rats via oxidation and ER stress stimulation. Accordingly, the peroxidation inhibitor N-acetyl-cysteine (NAC) and the eIF2α dephosphorylation blocker Salubrinal (Sal) were utilized to verify the toxic mechanism of PS-MPs. Our study could provide a better mechanical understanding of the health risks of early life exposure to MPs.

## 2. Materials and Methods

### 2.1. Materials

PS-MPs were obtained from the Tianjin Baseline ChromTech Research Centre (2.5 percent *w*/*v*, 10 mL) (Tianjin, China). The PS-MPs employed in the investigation have a diameter of 1 μm. Scanning electron microscopy (SEM) was performed to characterize the morphology of the PS-MPs (Regulus 8100, Hitachi, Japan). Spectroscopy dispersion was used to determine the diameter of the PS-MPs suspended in ultra-pure water (Zetasizer Nano ZS90, Malvern Instruments Ltd., Malvern, UK) and chemical components were analyzed by Fourier transform infrared spectroscopy (FTIR) (Fourier Transform Infrared Spectrometer, Nicolet iS50, Shanghai, China). Beyotime Biotechnology Co., Ltd. (Shanghai, China) and Macklin Reagents, Ltd. (Shanghai, China) provided the NAC and Sal, respectively.

### 2.2. Animals and Experiment Design

Twenty-four female Sprague Dawley (SD) rats (3 weeks old, 52 ± 3 g) were supplied by Nanchang University’s experimental animal center (Nanchang, China). Food and water were available to all the rats in the animal laboratory room *ad libitum*, which was kept at a consistent temperature (25 °C) and light (12 h dark/12 h light cycles). Four rats were kept per cage. All animal experiments followed the standards of the institutional animal care committee and were authorized by the Animal Care Review Panel (approval number 0064257; Nanchang University, Nanchang, China). After a week of acclimatization, all rats (4 weeks old, 70 ± 5 g) were freely categorized into three groups (*n* = 8): 0 (control), 0.5, and 2.0 mg/kg. The PS-MPs treatment groups received 0.5 and 2.0 mg/kg by oral gavage, once daily for 28 consecutive days, respectively, whereas the control group received the same volume of deionized water. The high dose employed here was determined by an earlier article that claimed PS-MPs significantly cause intestinal injury and intestinal barrier dysregulation in vivo at 2.0 mg/kg [28], which may promote PS-MPs to pass through the gut to distant organs. Moreover, the exposure levels employed here are based on the precautionary principle, which states that identifying the target machine is a prerequisite to evaluating the risk at environmentally relevant concentrations, according to a previous study [29]. An earlier study showed that oral gavage of 0.1 mg/d (about 1.5 × 10^6^ particles/day) [22] PS-MPs caused significantly impaired ovarian function in rats. In our experiment, rats weigh 200 g on average, thus a dose of 0.5 mg/kg/d corresponds to a daily intake of PS-MPs of 0.1 mg. Therefore, we believe that the dose of 0.5 mg/kg/d PS-MPs can cause reproductive toxicity. Additionally, 0.1–0.5 g of MPs may be consumed by humans per week on average in the world [30]. The average rat weighs 200 g in our experiment, with a daily intake of PSMPs of 0.2 mg and 0.5 mg, then a weekly intake of 2.8 mg and 1.4 mg MPs, respectively, both of which are within the acceptable range for human ingestion. Prior to usage, MP particles were disseminated in deionized water and subjected to 20 min of supersonic wave vibration to thoroughly suspend them. During the trial, the rats’ weight gain and associated presentations were monitored daily. All rats were sacrificed by cervical dislocation at PND 59, which already have full sexual maturity, and their organs were quantified. Blood was collected by eyeball extraction. The serum was isolated at 4000 rpm for 10 min and stored at −20 °C for 24 h. For further analysis, the serum and tissue specimens were frozen at −80 °C.

### 2.3. NAC and Sal Supplementation

After one week of acclimatization, thirty-two female rats were split into four categories: the control (Group C), 2.0 mg/kg PS-MPs (Group I), 2.0 mg/kg PS-MPs with NAC (Group II), and 2.0 mg/kg PS-MPs with Sal (Group III) treatment groups (*n* = 8). The rats in Group C were treated with deionized water, and the rats in the other three groups were administered 2.0 mg/kg through oral gavage. Once every two days, Groups II and III received 100 mg/kg NAC and 1.5 mg/kg Sal by intraperitoneal injection, respectively [25]. Half an hour following the end of the gavage operation, the intraperitoneal injection procedure was carried out. NAC and Sal exposure times were 14 days due to the 28-day length of the overall experimental period. Beyotime Biotechnology Co., Ltd. (Shanghai, China) and Macklin Reagents, Ltd. (Shanghai, China) provided the NAC and Sal, respectively. The rats in Groups C and I were intraperitoneally injected with the same volume of sterile saline. The weights of the rats were measured daily for 28 days. All rats were sacrificed by cervical dislocation at PND 59, which already have full sexual maturity. The procedures of blood sample collection and sample storage were the same as described above.

### 2.4. Staining with Hematoxylin and Eosin (H&E) and Histomorphometric Analysis

The right ovaries were fixed in Bouin’s solution for 24 h and then dehydrated in graded ethanol before being embedded in paraffin. The paraffin-embedded ovaries were sectioned at 5 μm thickness and flattened on a slide before being stained with H&E. Five ovarian samples from each group were used to produce H&E slices and one section per animal. The sections were sealed with a cover glass and filmed using an optical microscope (Nikon T-U optical microscope, Tokyo, Japan). The percentage of atretic oocytes in each area was recorded, and the ovarian eggs were categorized according to the findings of a previous study [31]. The oocyte in the primordial follicle is surrounded by a layer of three to six flattened pre-granulosa cells; the oocyte in the primary follicle is surrounded by a layer of expanded cells; the secondary follicles have one to two layers of granulosa cells that are slightly larger in diameter around the oocytes; the preantral follicles have a variety of layers of granulosa cells that are larger. The primary follicles, secondary follicles, and preantral follicles were classified as growing follicles. To count the atretic follicles, 5 non-overlapping visual areas were randomly chosen from each slice under the microscope and the sum represented the total number of atretic follicles in one ovarian sample. The present “atretic follicle ratio (%)” per animal = the total number of atretic follicles seen in all five visual areas/total number of follicles (atretic follicles plus non-atretic follicles) seen in the same visual fields.

The quantitative histomorphometric analyses were performed on rats’ ovaries according to Tassinari et al. [32]. Briefly, using one of the largest sections in a central position of the ovary, primary and secondary follicles, preantral follicles, and atretic follicles were counted in the whole ovarian section.

### 2.5. Analysis of Sex Hormones and Oxidative Stress

The serum levels of estradiol and progesterone were determined using ELISA kits (Shanghai YSRIBIO Industrial Co., LTD., Shanghai, China), and the tiers of the ROS stress indicators superoxide dismutase (SOD), catalase (CAT), and malondialdehyde (MDA) in the ovaries were determined using commercial kits (Jiancheng, Nanjing, China). Fresh ovarian samples weighed approximately 0.1 g and saline was added following the standard of adding 1 g of tissue to 9 mL of homogenate. After homogenizing tissues in an ice bath, 10% tissue homogenates were obtained. The supernatant was centrifuged at 5000 rpm for 15 min and stored at −80 °C for protein measurement. A commercial BCA Protein Assay Kit (Applygen Technologies Inc., Beijing, China) was used to quantify the tissue protein concentration. The manufacturer’s instructions were followed for all parameters.

### 2.6. Quantitative Polymerase Chain Reaction in Real-Time (RT-qPCR)

Liu et al. found that the relative fluorescent intensity of GSH in mouse oocytes was significantly decreased after PS-MPs exposure [18]. Therefore, we chose the gene *GSH* as one of the oxidative stress indicators in this study. We explored the ovarian injury caused by PS-MPs exposure via the PERK-ATF4-eIF2α-CHOP signaling pathway. The PERK-ATF4 pathway is critical for regulating the CHOP expression, with increased CHOP expression leading to cellular apoptosis [33]. CHOP regulates ER stress-induced apoptosis by boosting the transcription of the pro-apoptosis gene Bax and suppressing the production of anti-apoptosis Bcl-2 in the mitochondrial membrane [34]. Caspase-9, which can be activated by the imbalance of Bcl-2 and Bax, engages in the subsequent activation of Caspase-3 [35]. The total RNA was isolated from the ovaries using kits (TSINKE Biotechnology Co., Ltd., Beijing, China). Then, using a reverse transcriptase mixture (Takara PrimeScript RT reagent package (Cat#RR047A, Lot#AK2802), the RNA of each subject was retro-converted into cDNA. Primer Express Software (Applied Biosystems, Foster City, CA, USA) and Oligo Primer Analysis Software version 6.0 (Molecular Biology Insights, Inc.; DBA Oligo, Inc. Colorado Springs, CO, USA) were used to construct the primers (Table 1). General Biosystems Co., Ltd. was commissioned to manufacture the primers (Chuzhou, China). According to prior studies [36,37], 40 cycles of 5 s at 95 °C, 5 s at 95 °C, and 1 min at 60 °C were programmed into the AriaMx Real-time qPCR system (New York, NY, USA, Agilent, MY 19435252). Relative quantification of messenger RNA (mRNA) was determined using the 2-∆∆Ct method, with GAPDH as the housekeeping gene.

### 2.7. Immunohistochemical (IHC) Analysis

The ovarian tissue embedded in paraffin is sectioned, dewaxed with xylene, and hydrated. Sections were then repaired with antigen and immersed in 3% hydrogen peroxide for the elimination of endogenous peroxidase activity. Subsequently, the sections were blocked with 3% BSA for 30 min at room temperature, and interacted with the following primary antibodies: BIP (1:100, Proteintech, Wuhan, China, 11587-1-AP) and PERK (1:200, Proteintech, Wuhan, China, 24390-1-AP) at 4 °C overnight. These slices interacted with IgG (1:200), an HRP-labeled rabbit secondary antibody, for 50 min following 3 PBS washes. Light-microscopy examination of the sections is performed after staining with a fresh 3,3′-diaminobenzidine solution and hematoxylin counterstain at room temperature. Positive cells are dark brown. For immunohistochemistry quantification, the average optical density of six high-magnification areas in three rat ovaries was determined using ImageJ 6.0.

### 2.8. Assay for Immunofluorescence

The abundance of target proteins eIF2α and Caspase-3 in the ovaries was measured by immunofluorescence. Previously embedded ovarian tissue blocks in paraffin in this work were deparaffinized with xylene and rehydrated with gradient ethanol. The slides were immersed in EDTA antigen buffer (pH 8.0) to retrieve the antigens. Then, 3% BSA was added to the slides to block the non-specific binding. The sections were incubated overnight at 4 °C with proportionally prepared primary antibodies eIF2α (1:500, GB13572) or Caspase-3 (1:500, GB11532) and rinsed, then incubated with fluorescent secondary antibody (1:300, GB21303) for 50 min. After the sections were dried, DAPI (Servicebio, G1012 Wuhan, China) was applied to counterstain the nuclei. This was incubated for 10 min, followed by the addition of a tissue autofluorescence quencher for 5 min. The sections were then washed with water and then blocked. The DAPI-stained nuclei turn blue, and the matching fluorescein-labeled green light indicates a positive expression (DAPI UV excitation wavelength 330–380 nm, emission wavelength 420 nm, blue light; FITC excitation wavelength 465–495 nm, emission wavelength 515–555 nm, green light). Finally, three slides per animal were analyzed and the sections were observed under a microscope, and images were collected using fluorescent microscopy (Nikon-Ti-U fluorescence inverted microscope, Tokyo, Japan). ImageJ 6.0 was used to examine the average image quantum yield with background subtraction (National Institutes of Health).

### 2.9. Analytical Statistics

The experimental data are reported as mean ± SD, and the results were statistically analyzed using SPSS (Version 26.0). One-way and two-way analysis of variance (ANOVA) combined with Tukey’s multiple comparison tests were used to determine the significance of differences between groups. Differences were considered significant at *p* < 0.05.

## 3. Results

### 3.1. Characterization of PS-MPs

**Figure 1:**
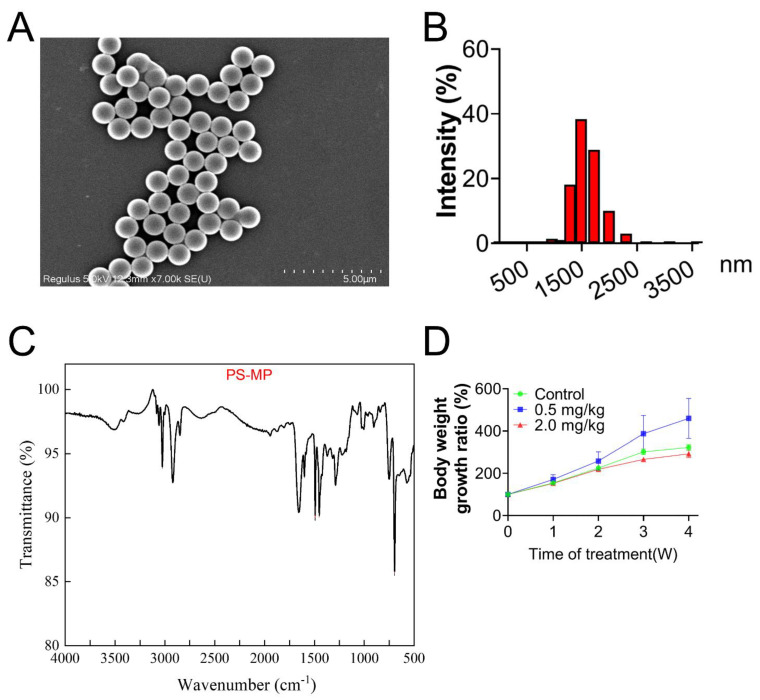
Characterization of PS-MPs by SEM, DLS, and FTIR. (**A**) PS-MP morphology (scale bar = 5 μm). (**B**) Hydrodynamic size distribution of PS-MPs. (**C**) FTIR image of PS-MP. (**D**) Body weight growth ratio.

All particles of PS-MPs approximated a spherical shape and were well-dispersed, as shown in the SEM image (Figure 1A). To measure the hydrodynamic size of PS-MPs, the solution of PS-MPs was diluted with deionized water to 0.1 mg/mL. PS-MPs presented a mean size of 1.5 μm (Figure 1B). Characteristics of PS-MPs displayed by FTIR confirmed that the chemical composition was polystyrene (Figure 1C), according to previous studies [38,39].

### 3.2. PS-MPs Exposure Has Negative Impacts on the Development of Follicles

**Figure 2:**
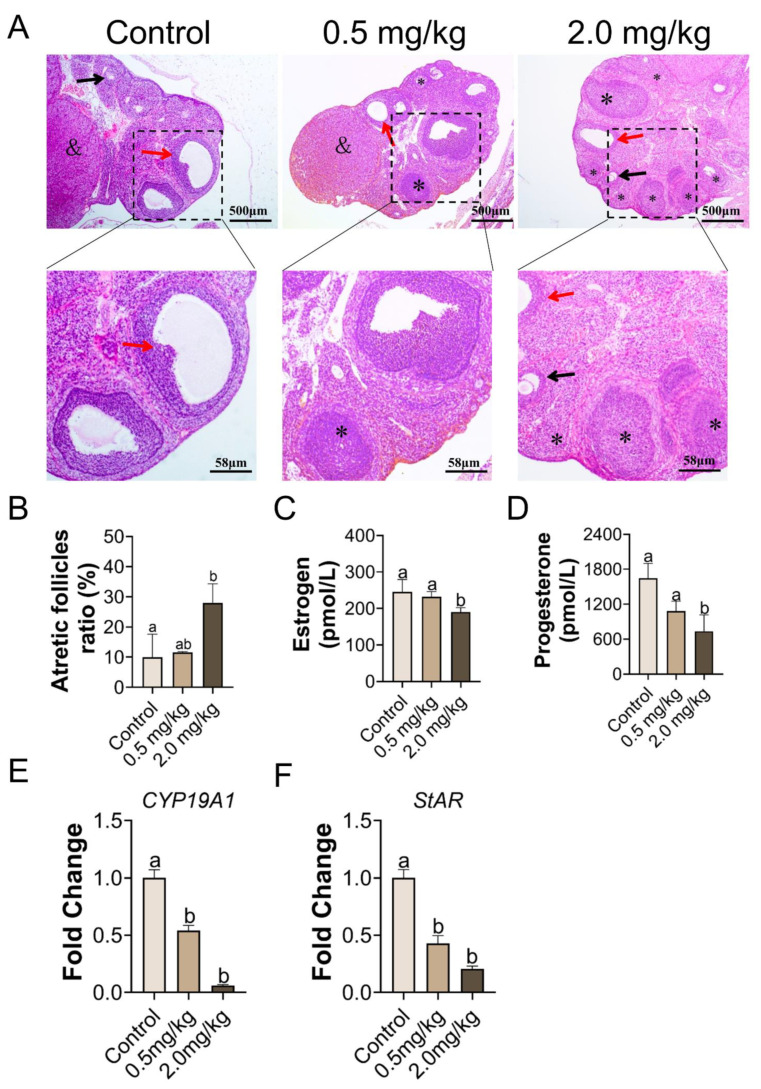
Histopathological alterations, serum hormone concentration, and gene expression relevant to hormonogenesis in response to PS-MPs. (**A**) The representative images of H&E stained ovary (black arrow: primary follicles, red arrow: cystic follicles, asterisk: atretic follicles, and: corpus luteum), *n* = 5 per group. (**B**) Atretic follicles ratio, *n* = 5 per group. (**C**,**D**) ELISA was used to assess the amount of estradiol and progesterone in serum. (**E**,**F**) qPCR detection the comparative mRNA expression of CYP19A1 and StAR as in ovary of rats, standardized to GAPDH, *n* = 3 per group. The information was presented as the mean ± SD. Different letters indicate significance.

As shown in Figure 2A, no obvious structural differences in follicles existed between the control and 0.5 mg/kg PS-MPs groups. However, the percentage of atretic follicles in the 2.0 mg/kg PS-MPs group was higher (*p* < 0.05, Figure 2B) than that in the other groups. Histomorphometric data were summarized in Table S1. The number of growing follicles significantly decreased at a dose of 2.0 mg/kg PS-MPs level. Furthermore, the 2.0 mg/kg PS-MPs group presented notably reduced serum levels of estrogen and progesterone compared with the control group (Figure 2C,D). No difference in findings was observed between the 0.5 mg/kg PS-MPs and control groups. Moreover, the PS-MPs group exhibited considerably lower levels of estrogen controller cytochrome P450 family 19 subfamilies A member 1 (CYP19A1) and steroidogenic acute regulatory protein (StAR) (Figure 2E,F). Nevertheless, the bodyweight growth ratio during the experiment showed no significant difference between the control and 2.0 mg/kg PS-MPs group except for a decreased trend in the 2.0 mg/kg PS-MPs group from the second week (Figure 1D). Likewise, the ovarian index in PS-MPs groups was lower than that in the control group, but had no significance (Figure S1A). In addition, we observed that the ovarian weight significantly declined after PS-MPs exposure compared with the control (Figure S2A).

### 3.3. Exposure to PS-MPs Culminated in Oxidative Response in the Ovary

**Figure 3:**
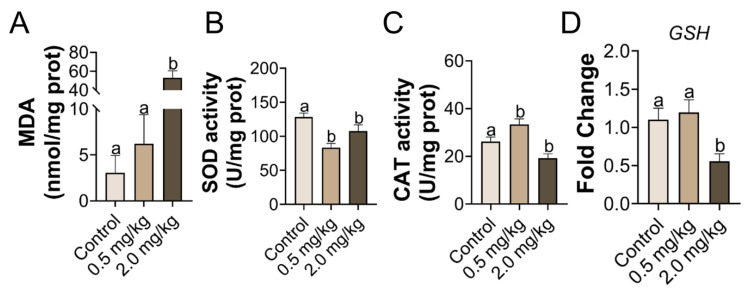
PS-MPs cause oxidative stress in the ovary. (**A**) The concentration of MDA, the antioxidant activity within SOD, and CAT (**B**,**C**). (**D**) The comparative mRNA expression of GSH in the ovary was analyzed. The information was presented as the mean ± SD. Different letters indicate significance. *n* = 5 per group.

The MDA levels were higher in the 2.0 mg/kg PS-MPs treatment group than in the control group (*p* < 0.05, Figure 3A). Compared with the control group, the antioxidant catalytic abilities of SOD and CAT were drastically reduced in the 2.0 mg/kg PS-MPs group (*p* < 0.05, Figure 3B,C). However, the results in the 0.5 mg/kg PS-MPs group were equivocal and contradictory on the activity of SOD and CAT (Figure 3B,C). The expression level of the antioxidative gene glutathione (GSH) decreased substantially in the 2.0 mg/kg group, as per RT-qPCR testing of the ovarian tissues (Figure 3D).

### 3.4. PS-MPs Consumption Led to ER Stress and Apoptosis in the Ovary

**Figure 4:**
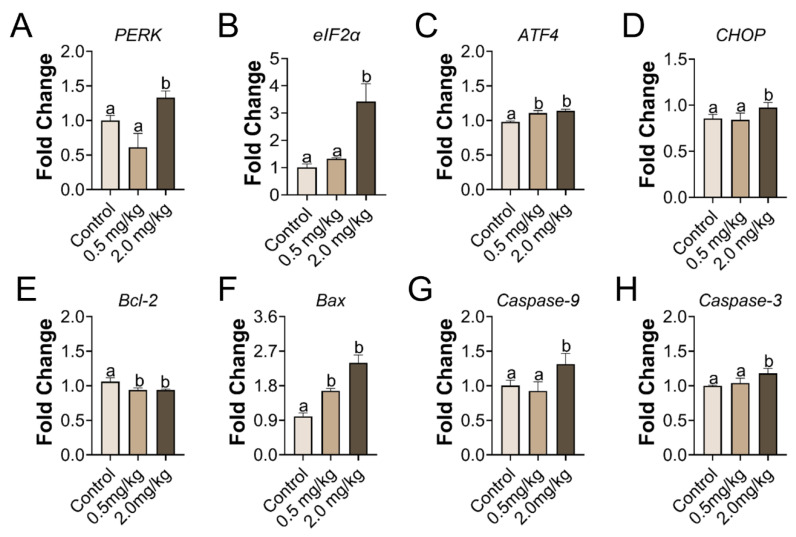
The mRNA expression of genes involved in ER stress and apoptosis in the ovary upon PS-MPs stimulation. (**A**–**D**) Relative expression levels of genes associated with ER stress, including *PERK*, *eIF2α*, *ATF4*, and *CHOP*. (**E**–**H**) Relative expression levels of genes associated with apoptosis, including *Bcl-2*, *Bax*, *Caspase-9*, and *Caspase-3*. The information was presented as the mean ± SD. Different letters indicate significance. *n* = 3 per group.

Gene transcription related to ER stress and apoptosis was investigated in the ovary. In the group treated with 2.0 mg/kg of PS-MPs, the ER stress genes *PERK* and *eIF2α* markedly increased. *CHOP* and *ATF4* were augmented. In addition, the induced apoptosis genes, such as *Bax*, *Caspase-9*, and *Caspase-3*, were strongly amplified in the 2.0 mg/kg PS-MPs group, but *Bcl-2* was greatly dysregulated (Figure 4). Relative expression of *ATF4*, *Bax* and *Bcl-2* in the 0.5 mg/kg PS-MPs group showed significance compared with the control group. No significant differences were found among the extra genes in the 0.5 mg/kg PS-MPs group.

### 3.5. PS-MPs Exposure Induced Ovarian Apoptosis Closely Associated with Oxidative Stress and ER Stress

**Figure 5:**
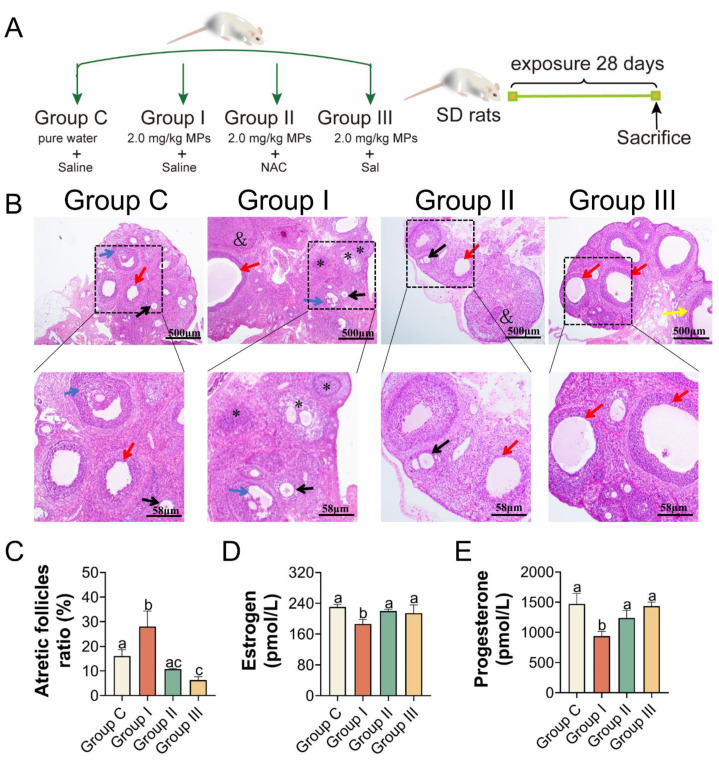
The effects of NAC and Sal treatment on atretic follicles ratio, serum hormone levels. (**A**) Format of the trial, groups, and interventions. (**B**) H&E staining in ovarian slices (black arrow: primary follicles, red arrow: cystic follicles, blue arrow: secondary follicles, yellow arrow: antral follicles, asterisk: atretic follicles, and: corpus luteum), *n* = 5 per group. (**C**) Atretic follicles ratio, *n* = 5 per group. (**D**,**E**) ELISA was used to assess the amount of estradiol and progesterone in serum. The information was presented as the mean ± SD. Different letters indicate significance.

**Figure 6:**
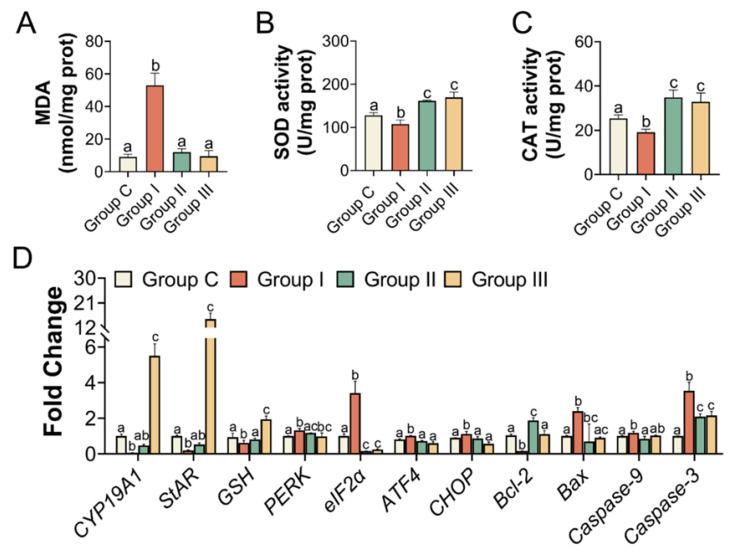
Oxidative stress indicators and relative expression of genes in the ovary. (**A**) The concentration of MDA, the antioxidant activity of SOD, and CAT (**B**,**C**), *n* = 5 per group. (**D**) The relative mRNA levels of oxidative stress, ER stress, apoptosis, and hormones in the ovary, *n* = 3 per group. Different letters indicate significance.

Based on the above results, 2.0 mg/kg of PS-MPs caused obvious ovarian damage, likely through oxidative stress and the PERK-eIF2α-ATF4 signaling pathway of ER stress. To further test the mechanism induced by PS-MPs, oxygen strain antagonist NAC and the eIF2α dephosphorylation blocker Sal were used. To evaluate the biological alterations after PS-MPs exposure, the ovarian index was recorded. Additionally, 2.0 mg/kg PS-MPs oral exposure induced a significant decrease in the ovarian index compared with Group C. There was an upward trend but no significance of the ovarian index with NAC and Sal treatment compared with the PS-MPs group (Figure S1B). Similarly, results of the absolute ovarian weight showed that the ER stress inhibitor Sal but not the NAC could effectively alleviate the loss of ovary weight induced by PS-MPs exposure (Figure S2B). As depicted in Figure 5B and histomorphometric data (Table S2), results showed that the number of ovarian atretic follicles increased and the number of growing follicles decreased in the PS-MPs group compared with the control. The proportion of atretic follicles in Group I was much lower than that in Group C. Following the NAC and Sal treatment, the percentage of atretic follicles decreased significantly compared to that in Group I (Figure 5C). The estrogen and progesterone levels in the serum were increased because of the effects of the NAC and Sal treatment (Figure 5D,E). The mRNA levels of CYP19A1 and StAR were elevated in Group I (Figure 6D).

**Figure 7:**
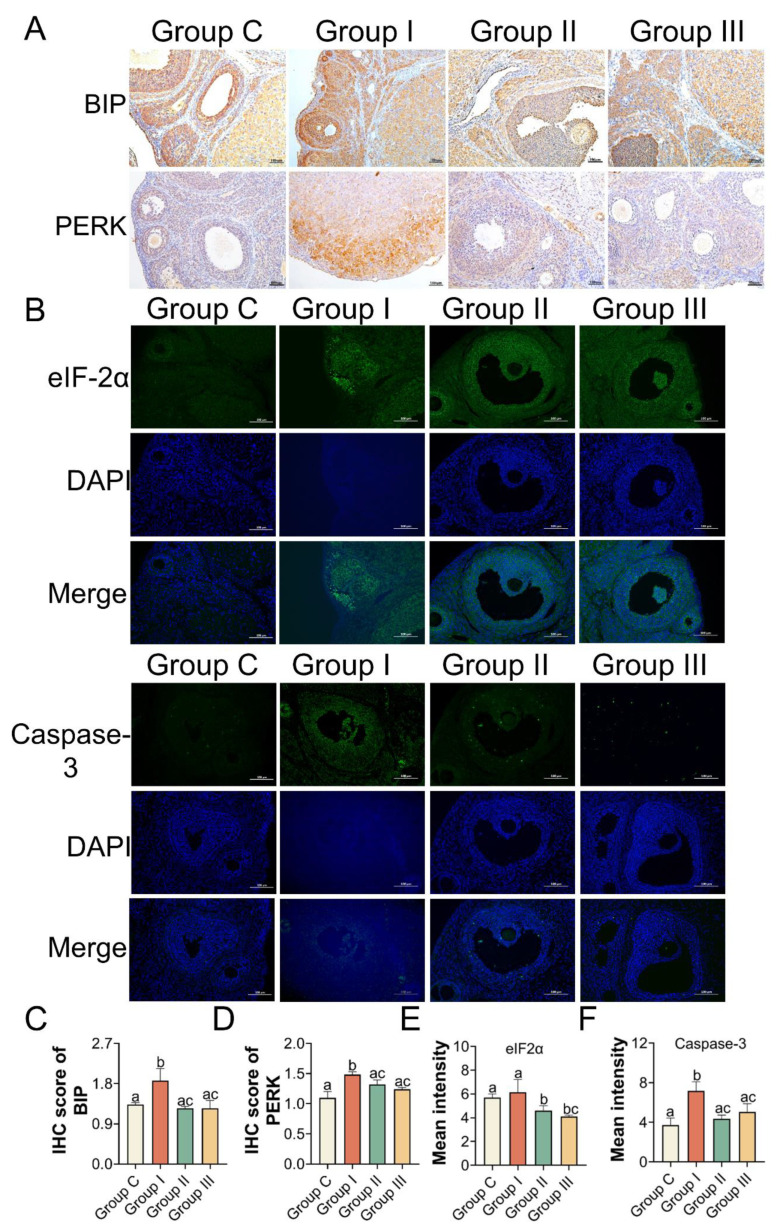
Protein activity of BIP, PERK, eIF2α, and Caspase-3 in ovaries after NAC and Sal treatment. (**A**) IHC images of BIP and PERK (scale bar = 100 μm). (**B**) eIF2α and Caspase-3. Blue: DAPI, green: eIF2α, and Caspase-3 (scale bar =100 μm). (**C**,**D**) IHC scores of BIP and PERK. (**E**,**F**) Mean intensity results. Different letters indicate significance. *n* = 3 per group.

**Figure 8:**
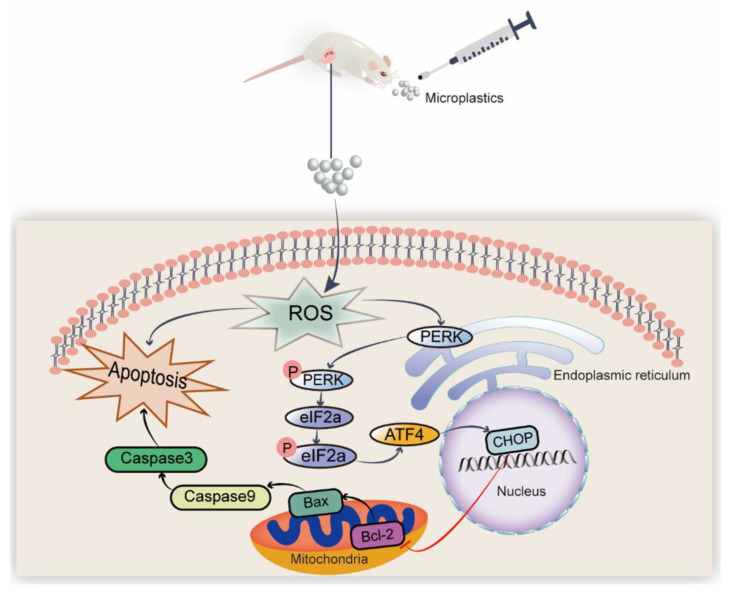
A proposed route of ovarian injury induced by PS-MPs administration. PS-MPs consumption enhanced the buildup in oxidative stress and then caused mitochondrial apoptosis by activating PERK-ATF4-eIF2α-CHOP molecular cascade. “↑” activation, “⊥” inactivation, and “P” phosphorylation.

In addition, the levels of antioxidant enzymes, such as SOD and CAT, in the ovaries were significantly elevated in Groups II and III compared to those in Group I. MDA levels were lower in Groups II and III than in Group I (Figure 6A–C). We also examined the transcriptional activity of the gene variants in ER stress and apoptosis. The mRNA expression levels in the NAC- and Sal-treated groups were similar to those in Group C (Figure 6D). IHC and immunofluorescence assays were performed to quantify the protein levels of BIP, PERK as well as eIF2α, and Caspase-3, separately, to further clarify the mechanism. According to the IHC staining, the IHC scores of BIP and PERK showed the same results that the levels of BIP and PERK were dramatically increased in Group I compared to Group C, which were both lowered (*p* < 0.05) with the NAC and Sal treatment (Figure 7A,C,D). The median intensity of eIF2α in the ovary in Group I exhibited an upward tendency but was not significantly different from that in Group C. Immunofluorescence analysis of Caspase-3 in Group I showed a marked increase compared to that in Group C. It presented a significantly lower mean intensity of eIF2α and Caspase-3 after NAC and Sal treatment than that in Group I (Figure 7B,E,F). Figure 8 illustrates the process of PS-MP-induced ovarian toxicity.

## 4. Discussion

Humans are ubiquitously in contact with MPs. The daily usage of plastic feeding bottles, water bottles, and medical syringes is the primary source of MPs for infants and children, causing harm to their bodies [40]. Moreover, the exposure of children to certain MPs components can affect their immune system components and functions, and even induce cancers, including ovarian cancer [41]. According to research, the health hazards of MPs to individuals, particularly children, should be taken seriously. In this study, a juvenile rat model was established to explore juvenile ovarian toxicity following MPs exposure.

The H&E sections showed that the number of ovarian atretic follicles increased and the number of preantral follicles decreased in the PS-MPs group compared with the control, indicating a change in ovarian structure. The ovary has two functions: endocrine and ovulation. The primary component of the ovary is the ovarian follicle, which provides the oocyte with a suitable environment for growth and development in preparation for ovulation and fertilization. Our findings showed that PS-MPs intake impaired follicle maturation. Estrogen and progesterone levels can affect follicle development by affecting granulosa cell (GC) growth and follicular fluid formation [42,43]. Therefore, in this investigation, the overall estrogen and progesterone concentrations in the 2.0 mg/kg PS-MPs group were generally lower than those in the control group. Our findings are consistent with those of previous research, showing that PS-MPs triggered fibrosis and death in the ovarian granulosa cells in adult rats [44]. Sex hormones, mainly estradiol and progesterone, are produced by the ovarian granulosa cells, which maintain oocyte development and follicle maturation [45]. Consequently, GC apoptosis is the main mechanism underlying follicular atresia and a decline in sex hormone levels. According to the literature, all the stages of follicular atresia are connected to the apoptosis of the GC and affect female fertility in adulthood by affecting ovulation [46]. Hou et al. discovered that 0.5 μm of PS-MPs may permeate the ovarian granulosa cells and trigger pyroptosis and apoptosis via the NOD-like receptor thermal protein domain associated protein 3-Caspase-1 signaling pathway in rats [47]. Previous studies demonstrated that 5 μm fluorescent PS-MPs accumulated in ovarian tissue after adult Wister rats exposure for 4 estrous cycles (24–26 days). Accumulation of corpus luteum and atretic follicles were higher than other types of follicles and stroma [22]. Fluorescence microscopy revealed the accumulation of microplastics (5–5.9 μm) in ovarian tissue [48]. MPs with a particle size of even 5 μm larger could accumulate in the ovaries. In the current work, we speculated that PS-MPs with diameter of 1 μm built up in the ovaries and induced ovarian damage. Microplastics were more likely to be internalized by cells when exposed to the environment. Additionally, internalization of cells was a crucial avenue for the transfer of microplastic particles into tissues, where they could have toxicological consequences that exert an impact on both human and environmental health [49]. Therefore, our observations suggested that PS-MPs exposure impaired follicle development and ovarian function through the increase in follicular atresia.

To further prove that the hormone synthesis was affected by PS-MPs exposure, we also analyzed the gene level. Steroid hormones play a crucial role in follicular atresia. Aromatase is the crucial enzyme facilitating the critical stage of estrogen production, and CYP19A1 is a member of the cyp450 gene superfamily that encodes it. Estrogen release deficits, GC condition aberrations, ovulation insufficiency, or even female fertility are mainly characterized by CYP19A1 disruption [50]. Furthermore, StAR is implicated in the regulation of the rate-limiting phase in hormone secretion and thus precipitates a permitting process in progesterone generation [51]. PS-MPs intake drastically reduced the CYP19A1 and the StAR expression compared with the control group, implying that PS-MPs consumption was deleterious toward hormonogenesis. PS-MPs also effectively diminished the StAR expression in the testicular tissue of rats [52]. Treatment with NAC and Sal reversed these effects. These results indicated that the mechanisms of MPs-induced ovarian toxicity may be associated with oxidative stress and ER stress.

Understanding the ovarian toxicity of PS-MPs may help extrapolate the risks to mammals. The ovary is vulnerable to oxidative damage due to the richness of unsaturated lipids. Studies have demonstrated that oxidative stress activation has negative effects on follicle development, oocyte maturation, and ovulation [53]. The predominant mechanism of PS-MPs poisoning is oxidative stress. PS-MPs produced apoptosis and fibrosis in the follicle oocytes of rats due to oxidative stress [44]. PS-MPs exposure caused ovarian inflammation in rodents by lowering the ER calcium ([Ca^2+^] ER) levels and increasing the ROS in oocytes [18]. MDA is a lipid peroxidation cumulative measure that implicitly represents the degree of overall tissue lipid peroxidation damage. In this study, the activity of antioxidant proteins SOD and GSH in the 2.0 mg/kg PS-MPs group was less than that of the control group, while the MDA content was notably higher. Nevertheless, Liu et al. obtained the opposite results of the level of MDA to our results, which might be caused by the decline of fatty acids in PS-MPs treated group in the previous study [18]. However, the results for the 0.5 mg/kg PS-MPs group could be attributed to body defenses. Previous research shows that 1.5 mg/d of PS-MPs causes ovarian damage, which is linked to oxidative stress, as measured by the spike in MDA and the decline in antioxidant enzyme activity [44]. Furthermore, the mRNA expression level of GSH declined. PS-MPs-induced ovarian damage is linked to oxidative stress, which is consistent with our findings. The outcomes of peroxidation antagonist NAC administration, which could appreciably lessen ovarian peroxidation, corroborate this hypothesis. Interestingly, ER stress inhibitor Sal also blocks the elevated oxidative stress levels caused by PS-MPs. Early studies provided evidence that NAC and Sal are bidirectional inhibitors [25,54]. Hence, our findings implied that oxidative stress and endoplasmic reticulum stress were linked.

Oxidative stress could induce ER stress. Liu et al. found that oxidative stress mediates the ER stress induced by microcystin-leucine arginine in the ovary of mice [55]. The high levels of ROS stimulated by external materials can induce oxidative stress, leading to ER dysfunction that activates unfolded protein response (UPR). We found that 2.0 mg/kg/d of PS-MPs induced the significant up-regulation of the PERK signaling pathway-related genes, PERK, eIF2α, ATF4, and CHOP, compared to the control. A recent study elucidated that ER stress activation affects follicular and oocyte health, which consequently plays a role in the etiology of a number of ovarian disorders following exposure to environmental pollution [26]. In this study, we speculated that PS-MPs activated the PERK-ATF4-CHOP signaling pathway of ER stress. The UPR activation by ER stress has three branches: inositol-requiring enzyme 1 (IRE1), PERK, and ATF6. PERK-eIF2α-ATF4 is one of the most important and earliest pathways. The oral delivery of PS-MPs enhances ER stress in the liver via the PERK signaling cascade [56]. On the basis of previous work, we proposed that MPs induced ovarian apoptosis through the activation of the PERK-eIF2α-ATF4-CHOP signaling pathway and oxidative stress.

ER stress is involved in ovarian GC apoptosis. In the 2.0 mg/kg/d condition, the PERK signaling pathway and the apoptotic genes were dramatically engaged. Therefore, our observations revealed that PS-MPs induced ovarian apoptosis was probably associated with the oxidative stress and activation of the PERK--eIF2α-ATF4-CHOP in juvenile rats.

To further verify the mechanism, rats from Groups II and III were intraperitoneally injected with cellular redox blocker NAC and eIF2α dephosphorylation inhibitor Sal after 2.0 mg/kg/d PS-MPs gavage. With inhibitor treatment, the atretic follicles ratio, levels of estrogen and progesterone in the serum, and the oxidative stress indicators of MDA, SOD, and CAT in the ovary tissues were corrected accordingly compared with the PS-MPs group. Sal can specifically inactivate eIF2a phosphatase complexes, boost eIF2a phosphorylation levels, lower translation rates, and restore ER homeostasis. The ovulatory dysfunction of obese mice treated with Sal was remedied, and their oocyte developmental competency improved [57]. Thus, reducing ER stress in the ovary could be a potential treatment method to replace hormone therapy. Treatment with NAC downregulated the expressions of PERK, eIF2α, ATF4, and CHOP compared to that in Group I, as supported by the IHC results of BIP and PERK, and immunofluorescence results of eIF2α and Caspase-3. Consequently, our results confirmed that PS-MPs consumption promoted ovarian apoptosis, which is likely attributed to oxidative stress-induced PERK-eIF2α-ATF4-CHOP transcriptional activation.

This study provides basic toxicological data to elucidate the impact of MPs on the reproductive system of young mammals. However, direct evidence of PS-MP being present in ovarian tissue was absent, and this study did not follow the impact of young mices’ exposure to MPs on fertility in adulthood. More evidence suggesting the PERK-eIF2α-ATF4-CHOP signaling pathway involving oxidative stress and apoptosis is lacking in our study, particularly in Western blot data. Thus, the impact of MPs on children’s health requires further investigation. Furthermore, appropriate and proportionate measures should be taken to reduce children’s exposure to MPs.

## 5. Conclusions

Summarily, our study indicates that PS-MPs could induce ovary apoptosis in juvenile rats through oxidative stress and activation of the PERK-eIF2α-ATF4 signaling pathway, resulting in the defective development of follicles, decreased production of estrogen and progesterone, decreased activity of SOD and CAT, and increased MDA in juvenile rats. Furthermore, a ROS scavenger NAC and eIF2α dephosphorylation inhibitor (Salubrinal) effectively attenuated the quality of follicles, improving estrogen and progesterone production and associated enzyme activity. Our findings highlight the negative impacts and mechanisms of PS-MPs on female juvenile reproduction, presenting new avenues for analyzing the health hazards of exposure to MPs for children. We urge more academics to study juvenile toxicity exposure to chemicals and to take additional steps to shield children from environmental pollution in light of our results.

## Figures and Tables

**Table 1:** Primers used for a quantitative real-time polymerase chain reaction.

Gene	Primer	Sequence (5′→3′)
*CYP19A1*	Forward	GGAAATCCACACTGTTGTTGG
	Reverse	TGAAGTTTTCCACCACTTTCAA
*GAPDH*	Forward	CTCATGACCACAGTCCATGC
	Reverse	TTCAGCTCTGGGATGACCTT
*StAR*	Forward	GAAAGCCAGCAGGAGAATGG
	Reverse	CACCTCCAGTCGGAACACCTT
*GSH*	Forward	ATCCCACTGCGCTCATGACC
	Reverse	AGCCAGCCATCACCAAGCC
*PERK*	Forward	GATACGGCATTTGGCTTGGG
	Reverse	CCCATGATTCTCGGCATCCA
*eIF2α*	Forward	AGCAATGGAGAAAATTTGCCTTGA
	Reverse	TCTGACCAGGAAGGACACCA
*CHOP*	Forward	GCAGCGACAGAGCCAAAATAA
	Reverse	CTGCTTTCAGGTGTGGTGGT
*BCL-2*	Forward	CTTTGAGTTCGGTGGGGTCA
	Reverse	CATCCCAGCCTCCGTTATCC
*Bax*	Forward	CGTCTGCGGGGAGTCAC
	Reverse	ATCTGTTCAGAGCTGGTGGG
*Caspase-3*	Forward	GCGTAAGGAAAGGAGAGGTG
	Reverse	ACAGACCAGTGCTCACAAGG
*Caspase-9*	Forward	GGCCTTCACTTCCTCTCAAG
	Reverse	GGACACAAGGATGTCACTGG
*ATF4*	Forward	AGTCTGCCTTCTCCAGGTGTTC
	Reverse	GCTGTCTTGTTTTGCTCCATCTT

## Data Availability

The data presented in this study are available in this article.

## Supplementary Materials

The following supporting information can be downloaded at: https://www.mdpi.com/article/10.3390/toxics11030225/s1, Figure S1. Alterations of the ovarian index induced by PS-MPs exposure. Figure S2. Absolute ovaries’ weight of female rats treated with PS-MPs. Table S1. Histomorphometric data of female rats treated with PS-MPs at a dose level of 0, 0.5, and 2.0 mg/kg per day during the juvenile period (PND 28-56). Table S2. Histomorphometric data of female rats treated with deionized water (Group C), PS-MPs (Group I), PS-MPs + NAC (Group II), and PS-MPs + Sal (Group III) during the juvenile period (PND 28-56).
