# Supplementary material for: Polystyrene Microplastics Induced Ovarian Toxicity in Juvenile Rats Associated with Oxidative Stress and Activation of the PERK-eIF2α-ATF4-CHOP Signaling Pathway
Source: Toxics. 2023 Feb 27;11(3):225. doi: 10.3390/toxics11030225 (PMC10057489; doi:10.3390/toxics11030225)
Supplement: Supplementary file 1 [file toxics-11-00225-s001.zip › toxics-2007642-supplementary.pdf]

toxics

MDPI

Supplementary Materials: Polystyrene Microplastics Induced Ovarian Toxicity in Juvenile Rats Associated with Oxidative Stress and Activation of the PERK-eIF2α-ATF4-CHOP Signaling Pathway

Wanzhen Wang, Jiafu Guan, Yueying Feng, Shanji Liu, Yu Zhao, Yuanyuan Xu, Hengyi Xu, Fen Fu

Table S1. Histomorphometric data of female rats treated with PS-MPs at a dose level of 0, 0.5, and 2.0 mg/kg per day during the juvenile period (PND 28–56).

Organ	Observation	Dose mg/kg per day		
		Control (n = 5)	0.5 (n = 5)	2.0 (n = 5)
Ovary	Number of atretic follicles	3.0 ± 2.0	5.7 ± 3.2	9.0 ± 2.6*
	Number of growing follicles	13.3 ± 2.5	15.3 ± 1.5	9.3 ± 2.5*

*$p < 0.05$ compared with control.

Table S2. Histomorphometric data of female rats treated with deionized water (Group C), PS-MPs (Group I), PS-MPs + NAC (Group II), and PS-MPs + Sal (Group III) during the juvenile period (PND 28–56).

Organ	Observation	Group C (n = 5)	Group I (n = 5)	Group II (n = 5)	Group III (n = 5)
Ovary	Number of atretic follicles	6.3 ± 1.5	13.2 ± 2.0	10.8 ± 1.6	7.2 ± 0.8
	Number of growing follicles	20.5 ± 1.8	12.7 ± 2.5*	15.5 ± 2.6	15.0 ± 3.0

*$p < 0.05$ compared with Group C.

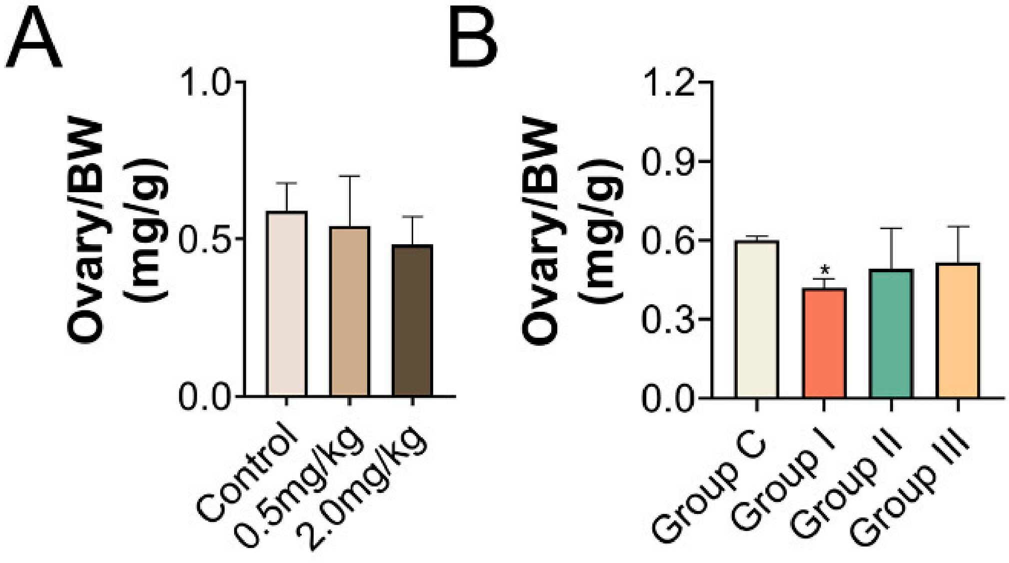

Figure S1. Alterations of the ovarian index induced by PS-MPs exposure. (A) The ovarian index between the control, 0.5 mg/kg and 2.0 mg/kg group. (B) The ovarian index between Group C, Group I, Group II, and Group III. *$p < 0.05$, compared with Group C, Data is presented as mean ± SD.

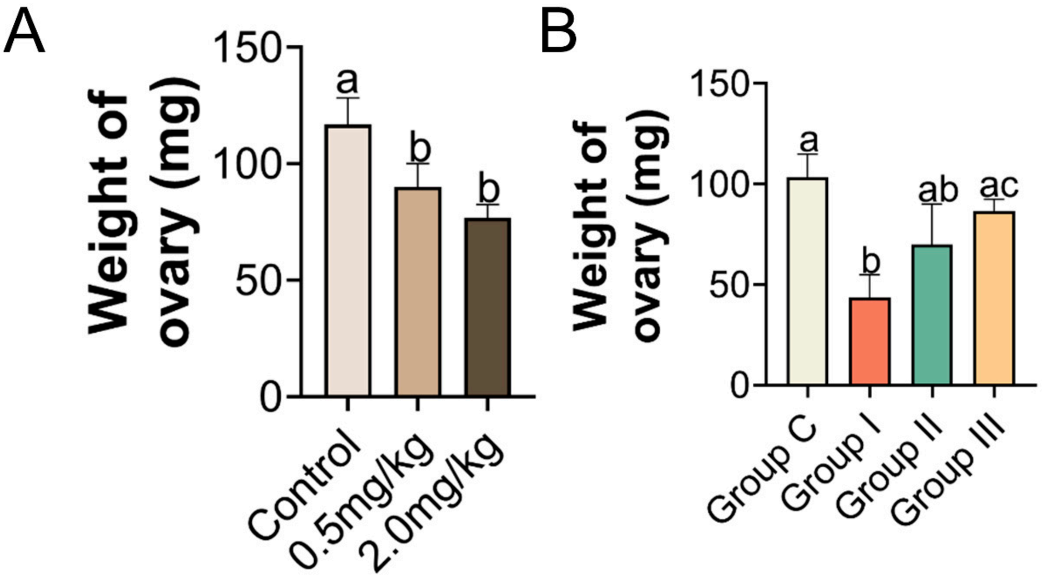

Figure S2. Absolute ovaries' weight of female rats treated with PS-MPs. (A) Weight of ovary (mg) at the PS-MPs dose levels of 0, 0. 5, and 2.0 mg/kg per day. (B) Weight of ovary (mg) between Group C, Group I, Group II, and Group III. Different letters between groups indicate significance.
